# Mechanical Properties and Fracture Behavior of Hot Isostatically Pressed TiC/TC4 Composites

**DOI:** 10.3390/ma18245529

**Published:** 2025-12-09

**Authors:** Zhiyu Sun, Jinyi Duan, Xiang Wu, Xiaofei Mo, Hai Nan, Jingchao Xu, Ao Fu, Yuankui Cao, Bin Liu

**Affiliations:** 1 Cast Titanium Alloy R&D Center, Beijing Institute of Aeronautical Materials, Beijing 100095, China; sunzy525@126.com (Z.S.);; 2 State Key Laboratory of Powder Metallurgy, Central South University, Changsha 410083, China

**Keywords:** titanium matrix composites, hot isostatic pressing, mechanical properties, fracture behavior

## Abstract

Titanium matrix composites (TMCs), characterized by low density, high strength, and excellent high-temperature mechanical properties, are becoming preferred materials for key components in aerospace engines. However, conventional casting methods for preparing TMCs often encounter issues such as composition segregation and coarse reinforcement phases, hindering their engineering application. In this study, we fabricated TiC/TC4 titanium matrix composites via hot isostatic pressing (HIP). The composites exhibited room-temperature tensile strength of 1058 ± 8 MPa, yield strength of 958 ± 12 MPa, and total elongation of 17.0 ± 0.5%. Furthermore, the TiC/TC4 composites demonstrated favorable high-temperature mechanical properties, with a tensile strength of about 500 MPa at 600 °C. Investigation into plastic deformation and fracture behavior revealed that at room temperature, tensile cracks initiated predominantly around the reinforcing TiC particles, whereas at high temperatures, cracks primarily originated within the matrix. The strengthening mechanisms of the TiC particle-reinforced TC4 composites included particle strengthening, solid solution strengthening, and load-transfer strengthening. Additionally, the precipitation of nano-acicular secondary α (αs) phases within the β phase during high-temperature tensile deformation was observed, contributing to the superior high-temperature mechanical performance of the composites.

## 1. Introduction

Titanium matrix composites (TMCs) combine the excellent toughness of the titanium alloy matrix with the high hardness of ceramic phases [1]. They also possess desirable properties such as low density, high-temperature resistance, creep resistance, high specific strength, and good corrosion resistance, granting them broad application prospects in components like blades and nozzles for advanced aerospace engines [2]. Commonly used reinforcement phases in TMCs currently include ceramic particles like TiC, TiB, SiC, as well as graphene [3,4,5]. These reinforcements can enhance the mechanical properties of the material while adding other functional characteristics through the second phase, significantly increasing the application value of TMCs. Based on the distribution characteristics of the reinforcement, TMCs can be classified into continuously reinforced and discontinuously reinforced composites [1]. Discontinuously reinforced TMCs are simpler to fabricate and have more controllable microstructures, making them more suitable for engineering applications [6]. For instance, Qiu [7] incorporated TiC particles and TiB whiskers as reinforcements into an IMI834 titanium matrix, respectively, and investigated their high-temperature properties. The results indicated that TiC particles provided better high-temperature strengthening effects than TiB whiskers. Lv [8] prepared Ti6Al4V (TC4) composites containing 1% short carbon fibers (CFs) via powder metallurgy. High-temperature tensile tests at 700 °C showed that the yield strength of the 1% CFs/TC4 composite was 74% higher than that of the TC4 matrix, demonstrating superior high-temperature mechanical performance.

Although conventional casted TMCs offer simple processes and low cost, the resulting products often exhibit coarse microstructures and are prone to defects such as porosity, inclusions, cracks, and segregation, which adversely affect the mechanical properties of the composites. Furthermore, TC4 has poor subsequent machinability, leading to severe tool wear and extremely high part manufacturing costs [9,10]. Additive manufacturing (AM) is an advanced near-net shaping process for fabricating TC4 and TMCs with complex geometries, which can improve raw material utilization and result in fine grains [11,12]. Li [13] prepared nano-TiBw reinforced TC4 composites using selective electron beam melting (SEBM). The TiBw nanoparticles refined the grains and hindered dislocation motion, enabling the TiBw/TC4 composite to maintain tensile strength comparable to that of TC4 at 500 °C when tested at 550 °C, while the elongation increased by 94%. However, the high cost and size limitations associated with this method restrict its widespread engineering application [14]. Powder metallurgy is currently a mainstream method for preparing TMCs, including spark plasma sintering (SPS), self-propagating high-temperature synthesis (SHS), spray deposition, etc. Among these, SPS is widely studied due to its rapid heating rate, short sintering time, uniform product grains, easily controllable microstructure, and the ability to produce materials with high density and good performance. For example, Dong [15] fabricated (TiC + Ti_2_Cu)/TC4 composites with a dual-scale micro-nano network structure via SPS, featuring micron-sized TiC particles distributed at grain boundaries and nano-Ti_2_Cu particles precipitated at α/β phase boundaries. The introduction of these reinforcement phases, combined with grain refinement, particle strengthening, and precipitation strengthening, synergistically increased the tensile strength of the composite by 32.2% compared to TC4. Nevertheless, the SPS process suffers from low thermal efficiency, high energy consumption, high cost, and is limited to producing small-sized TMCs, thus restricting its use in manufacturing large engineering components. The hot isostatic pressing (HIP) method utilizes near-net shaping of powders within specially shaped containers, enabling the production of large, complex-shaped products. This showcases significant potential for the industrial-scale production of TMCs. Cao [16] successfully prepared bulk graphene-reinforced TMCs using HIP. The addition of 0.5 wt.% graphene nanoflakes (GNFs) significantly enhanced the strength of the TMCs without compromising ductility, demonstrating the reliability of the HIP method. Song [17] characterized the room-temperature tensile fracture of TP-650 TMCs prepared by a pre-treatment melt process and found that the fracture was controlled by the brittle fracture of TiCp, followed by ductile fracture of the titanium matrix. Fruhauf [18] reached similar conclusions from their characterization of the room-temperature tensile fracture of HIPed 15% TiCp/Ti. Evidently, current research on the fracture behavior of TMCs, particularly their high-temperature fracture behavior, remains limited. Therefore, studying the fracture behavior of such materials is crucial for promoting the efficient design of high-performance HIPed TMCs.

In this study, we prepared in situ TiC particle reinforced Ti-6Al-4V (TC4) composites using the hot isostatic pressing method. Their microstructures and room-/high-temperature mechanical properties were tested. The differences in crack initiation and propagation behavior during tensile deformation at different temperatures were analyzed, and the strengthening mechanisms of TiC particles at various temperatures were discussed.

## 2. Materials and Methods

**Figure 1:**
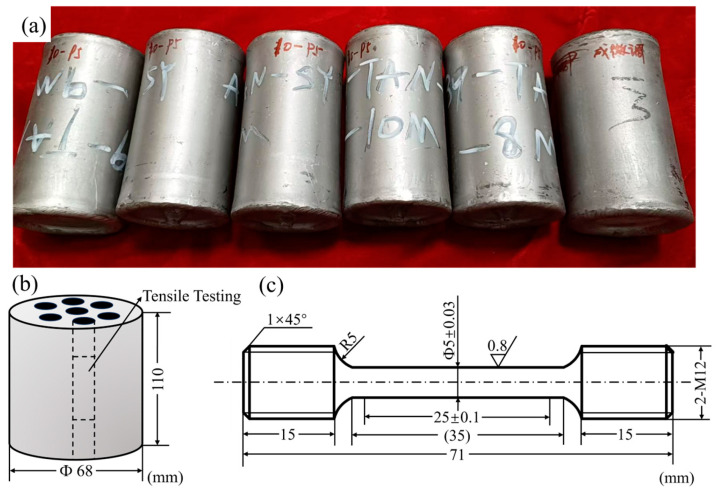
HIPed TiC/TC4 composite: (**a**) Billets figure; (**b**) Schematic illustrating tensile specimen extraction locations; (**c**) Tensile specimen geometry schematic.

Spherical TiC/TC4 composite powders, fabricated via the plasma rotation electrode process (PREP), were used as the starting materials. The powders were sieved to obtain a particle size range of 50–120 μm. These sieved powders were then loaded into a stainless-steel canister, which was subsequently evacuated, sealed by welding. The sealed canister underwent hot isostatic pressing (HIP) at a temperature of 1150 °C under a pressure of 160 MPa for a holding time of 2 h. Following the HIP cycle, the stainless-steel canister was removed via wire electrical discharge machining (EDM) to obtain the fully dense TiC/TC4 composite billet. The HIPed TiC/TC4 composite billets (dimensions: Φ68 × 110 mm) are presented in Figure 1a.

The phase composition, elemental distribution, and microstructure of the HIPed TiC/TC4 composite were characterized using scanning electron microscopy (SEM, Quanta FEG250, FEI, Portland, OR, USA, operated at 20 kV with a resolution of 3.0 nm) and electron backscatter diffraction (EBSD, Helios Nano Lab G3 UC dual-beam microscope system, FEI, Portland, OR, USA, operated at 20 kV with a resolution of 5.0 nm). The rod cross section was examined for microstructural characterization. Prior to these analyses, the specimens were successively ground using 400, 800, 1000, and 2000-grit SiC abrasive papers and then mechanically polished with an OPS solution. Specimen preparation for EBSD analysis included an additional electropolishing step. Electropolishing was conducted at a voltage of 30 V for 150 s at approximately −30 °C, using an electrolyte consisting of 6% perchloric acid, 34% butanol, and 60% methanol (by volume). Transmission electron microscopy (TEM) analysis was performed on a Tecnai G2 F20 microscope. Thin foils for TEM observation were prepared using a Tenupol-5 electropolishing unit from discs measuring 3 mm in diameter and 50 μm in thickness. A similar electrolyte to that used for EBSD was employed, with electropolishing conducted at 30 V and temperatures between −25 °C and −30 °C. Room and high-temperature tensile tests were carried out on a universal testing machine (MODEL-AGS-X-50kN, Shimadzu, Kyoto, Japan) at a constant strain rate of 1 × 10^−3^ s^−1^ to determine the ultimate tensile strength (UTS), yield strength (YS), and elongation (EL). Tensile testing was conducted at 600 °C and 650 °C to evaluate the high-temperature performance of the composite. Figure 1b illustrates the extraction locations of tensile specimens from the processed billet, while Figure 1c details the specimen geometry compliant with the ASTM E8 standard [19]. For each testing condition, three specimens were tested to ensure statistical reliability. The reduction of area (RA%) for tensile specimens was calculated in accordance with ASTM E8 using: $RA\%=\frac{\left(A_{0}-A_{f}\right)}{A_{0}}\times 100\%$, where *A*_0_ = Original cross-sectional area (mm^2^); *A_f_* = Minimum cross-sectional area at fracture (mm^2^).

## 3. Results and Discussion

**Figure 2:**
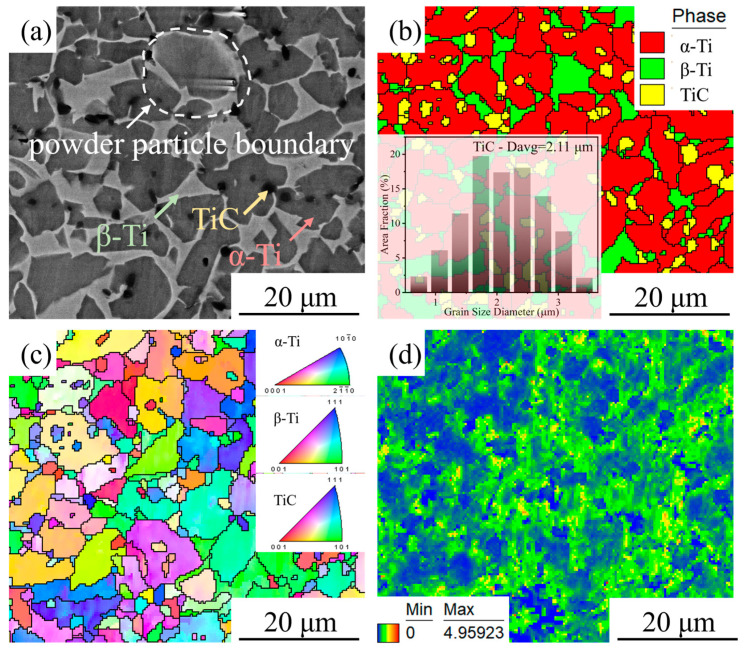
Microstructure of the HIPed TiC/TC4 composite: (**a**) SEM image; (**b**) Phase map; (**c**) Inverse pole figure (IPF) map; (**d**) Kernel average misorientation (KAM) map.

Figure 2a shows the scanning electron microscopy (SEM) micrograph of the HIPed TiC/TC4 composite. The matrix exhibits a typical α+β dual-phase microstructure, with the dark grey α phase presenting an approximately equiaxed morphology distributed within the light grey β phase. The TiC particles, appearing black in the SEM image, are predominantly located at grain boundaries, while a small fraction are precipitated within the grain interiors. These observations are consistent with previously reported findings [20]. Figure 2b–d presents the EBSD analysis results of the composite: the phase map, inverse pole figure (IPF) map, and kernel average misorientation (KAM) map, respectively. The phase map confirms the coexistence of α-Ti, β-Ti, and TiC phases within the material, with the TiC particles having an average size of 2.1 μm. These TiC particles are dispersed uniformly in the matrix without significant agglomeration. The IPF map indicates random crystallographic orientations of the grains, which is characteristic of the relatively isotropic nature of materials fabricated by HIP. The KAM map revealed a relatively uniform distribution of dislocations within the Ti matrix. Notably, dislocations were scarcely observed within the TiC particles themselves, and no significant stress concentration zones were evident, suggesting the production of a composite with a homogeneous microstructure via the HIP process.

**Figure 3:**
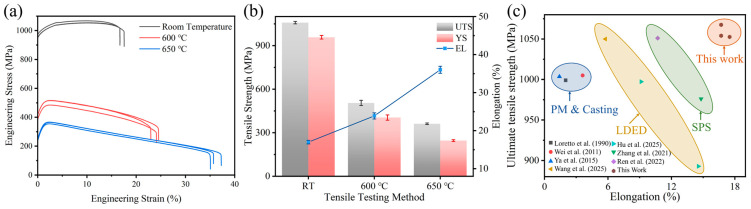
Mechanical properties of the HIPed TiC/TC4 composite: (**a**) Engineering stress–strain curves; (**b**) Comparison of mechanical properties under different temperatures; (**c**) Comparison of room-temperature tensile data with other fabrication processes [4,21,22,23,24,25,26,27].

The results of the room-temperature and high-temperature tensile tests for the TiC/TC4 composite are presented in Figure 3a,b. As the tensile testing temperature increased, the ultimate tensile strength (UTS) of the HIPed TiC/TC4 composite gradually decreased from 1058 ± 8 MPa (at room temperature, RT) to 505 ± 18 MPa (at 600 °C) and 362 ± 5 MPa (at 650 °C). Conversely, the total elongation (EL) of the composite increased from 17.0 ± 0.5% (RT) to 23.9 ± 0.8% (600 °C) and 36.0 ± 0.9% (650 °C), while uniform strain decreased sharply. RA% for optimal tensile specimens was quantified as 19.8% at room temperature, 31.5% at 600 °C, and 44.8% at 650 °C.

Figure 3c compares the room-temperature tensile properties of TC4-based composites fabricated by different processes. According to published data [21,22,23], TiC/TC4 composites prepared by casting and conventional powder sintering methods generally exhibit lower strength and toughness, with tensile strengths around 1000 MPa and elongations of only about 3%. The properties of composites produced via laser directed energy deposition (LDED) are highly influenced by process parameters and the type of reinforcement [24]. For instance, LDED-fabricated TiC/TC4 composites can achieve a room-temperature UTS of 1050 MPa, but typically suffer from low elongation. Furthermore, the overall mechanical performance of (TiB + TiC)/TC4 composites made by LDED was inferior to that of the HIPed TiC/TC4 composite in this work [25]. This is attributed to the inhomogeneous distribution of TiC particles along the deposition direction and changes in particle morphology during LDED solidification. Stress relief in these LDED materials occurs primarily through the fracture of the inhomogeneously distributed brittle eutectic TiC, thereby degrading the mechanical properties [24]. In contrast, composites reinforced with graphene and TiC fabricated via SPS demonstrate more uniform microstructures and significantly improved interfacial bonding, dislocation hindrance, and grain refinement [4,26]. Consequently, the yield strength and ultimate strength of these SPS composites are enhanced. The TiC/TC4 composite obtained in this work possesses exceptionally high comprehensive room-temperature mechanical properties. This is attributed to the finely dispersed TiC particles within the matrix and the relatively uniform equiaxed α+β dual-phase microstructure. The avoidance of stress concentration caused by inhomogeneous TiC distribution significantly improves the composite’s ductility. Moreover, compared to the TiB/TC4 composite also prepared by HIP [27], the present TiC/TC4 composite exhibits comparable high-temperature strength but better ductility. The suppressed RA% at RT correlates with dislocation pinning by TiC particles, which constrains plastic deformation. Conversely, elevated RA% values at high temperatures signify thermally activated dislocation slip/cross-slip mechanisms, enhancing material ductility. In summary, the HIP process enables TiC/TC4 composites with excellent combined mechanical properties at both room and elevated temperatures.

**Figure 4:**
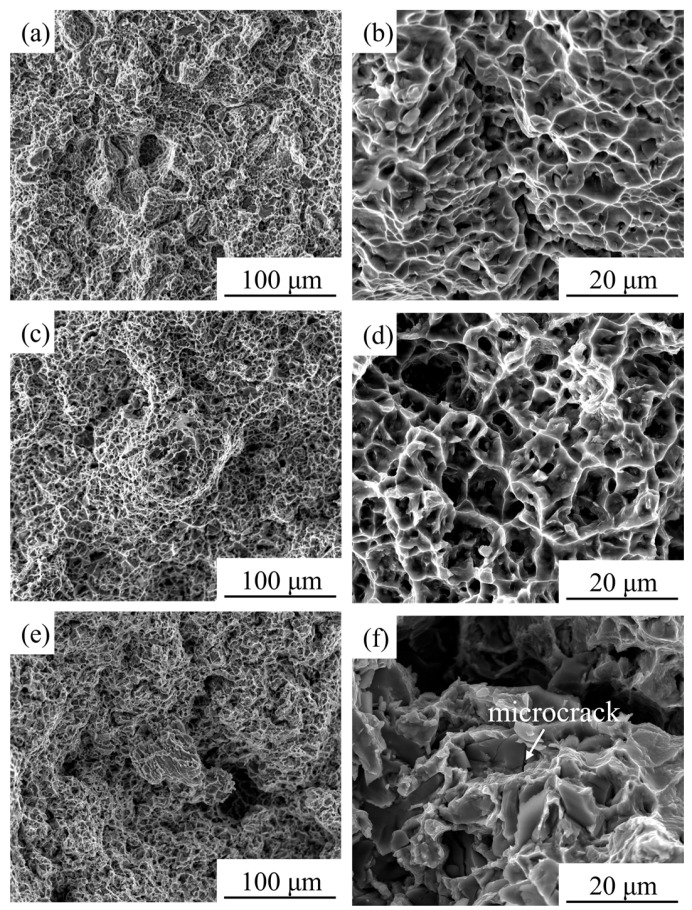
SEM images of tensile fracture surfaces: (**a**,**b**) RT; (**c**,**d**) 600 °C; (**e**,**f**) 650 °C.

Figure 4a,b shows the tensile fracture surfaces of the HIPed TiC/TC4 composite tested at room temperature. The high-magnification SEM images revealed intact, undeformed TiC particles, indicative of a particle pull-out fracture mechanism. The matrix region exhibited small and shallow dimples, characteristic of ductile fracture. Figure 4c,d and Figure 4e,f presents the fracture surfaces of specimens tested at 600 °C and 650 °C, respectively. The number of intact TiC particles observed on the high-temperature fracture surfaces decreases, and their edges appear blurred, which is related to the certain deformation capability of TiC particles at elevated temperatures. As the temperature increases, the average dimple diameter increased from 3 μm (RT) to 5 μm (600 °C) and 8 μm (650 °C), while the dimple depth in the matrix exhibited a non-monotonic variation, initially rising before declining. Fractographic analysis of high-temperature tensile specimens revealed distinct damage evolution: At 600 °C, the fracture surface exhibited predominant ductile rupture morphology characterized by high-density deep dimples. Conversely, the 650 °C specimen demonstrated a transitional ductile-brittle failure mode, showing reduced dimple quantity and flatter dimples, accompanied by increased microcracks and localized quasi-cleavage facets. Gurson’s damage mechanics model establishes a critical RA% threshold of 30% for fracture mechanism transition [28]: below this value, material failure is predominantly governed by interface decohesion, manifesting as synergistic quasi-cleavage and intergranular fracture modes; above 30% RA%, void nucleation and growth mechanisms dominate, generating dimpled rupture surfaces with localized shear bands. This theoretical framework aligns precisely with our fractographic analysis.

**Figure 5:**
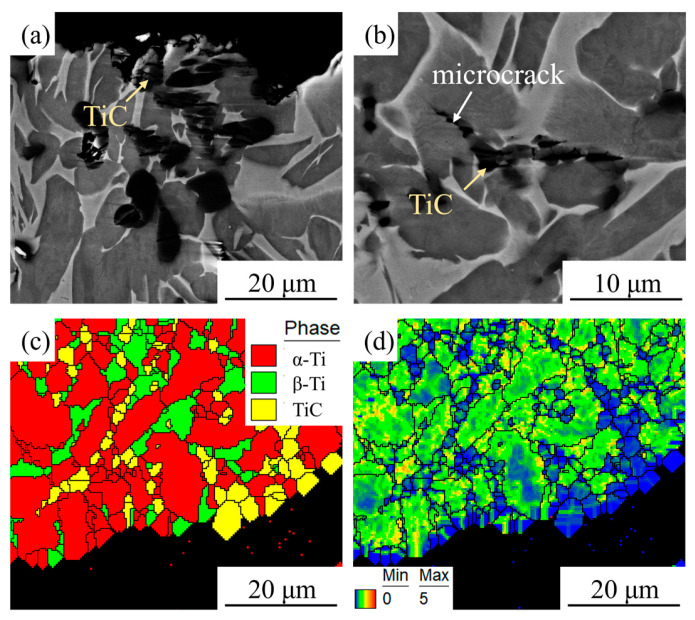
Microstructure near the room-temperature tensile fracture of the HIPed TiC/TC4 composite: (**a**) SEM image of the fracture zone; (**b**) SEM image of the microstructure near the fracture; (**c**) Phase map of the fracture zone; (**d**) KAM map of the fracture zone.

Figure 5a,b shows SEM images of the microstructure near the room-temperature tensile fracture of the HIPed TiC/TC4 composite. A considerable number of TiC particles were present near the fracture surface. Higher-magnification observation of the regions surrounding these TiC particles revealed the formation of microcracks around them, while almost no microcracks were observed within the matrix. This indicates that, under room-temperature conditions, TiC particles are the primary initiators of fracture microcracks, which subsequently propagate into the matrix, leading to the macroscopic fracture of the composite. This finding aligns with conclusions from previous studies [17]. Figure 5c,d presents EBSD analysis results of a phase map and the corresponding KAM map of the fracture zone. The distribution of TiC particles on the fracture surface is consistent with the SEM observations. Combined with the KAM map, it is evident that dislocations were scarcely present within the TiC particles, whereas the Ti matrix adjacent to the TiC particles exhibited a high dislocation density. This suggests that dislocations accumulate around the TiC particles during tensile deformation, contributing to the high strength of the TiC/TC4 composite. The internal residual stress within the fractured grains is relatively low, which is associated with the release of internal stress following grain fracture.

**Figure 6:**
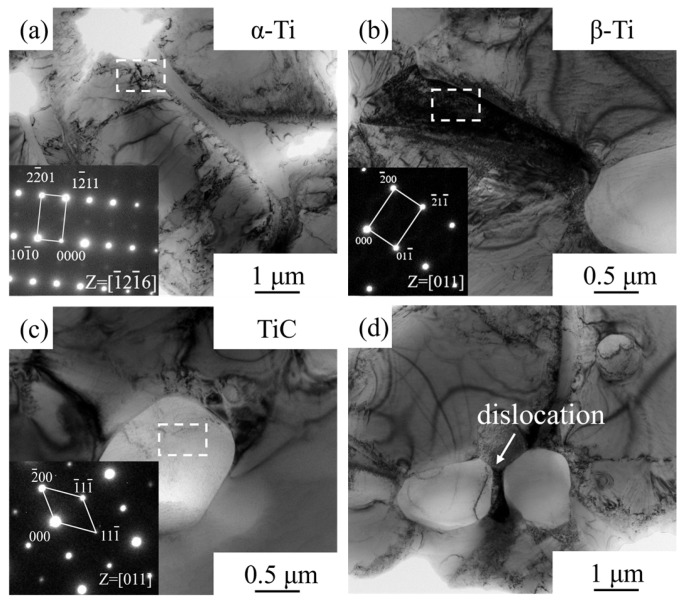
TEM characterization of the microstructure near the room-temperature tensile fracture of the HIPed TiC/TC4 composite: (**a**) α-Ti phase; (**b**) β-Ti phase; (**c**) TiC phase; (**d**) Bright-field image of the region between TiC particles.

TEM analysis was conducted on the microstructure near the room-temperature tensile fracture of the HIPed TiC/TC4 composite, with results shown in Figure 6. Figure 6a–c presents bright-field (BF) images characterizing the three constituent phases. Selected area electron diffraction (SAED) patterns were obtained from the areas marked by dashed lines in each image, shown in the lower-right corners. Consistent with prior analysis, the material consisted of α-Ti, β-Ti, and TiC phases. The dislocation density within the Ti matrix was significantly higher than that within the TiC particle reinforcement. Figure 6d is a BF image of two adjacent TiC particles. The region between the particles contains a high density of dislocations and experiences high micro-regional stress, making it prone to become a site for microcrack initiation and subsequent fracture. The micron-sized TiC particles effectively hinder dislocation motion while also contributing to strengthening through load transfer. Furthermore, studies have indicated that a portion of the carbon can dissolve into the matrix from TiC particle reinforcements, exerting a solid solution strengthening effect [29,30]. The synergistic operation of these strengthening mechanisms significantly enhances the strength of the HIPed TiC/TC4 composite at room temperature.

**Figure 7:**
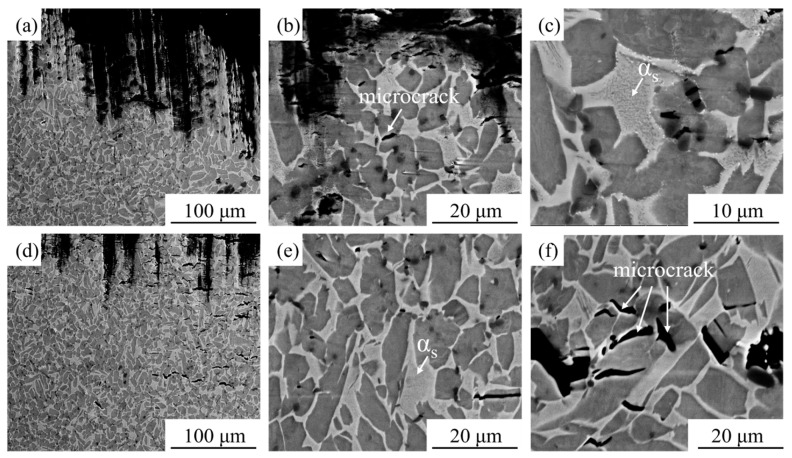
SEM images of the microstructure near the high-temperature tensile fracture surfaces of the HIPed TiC/TC4 composite: (**a**–**c**) 600 °C; (**d**–**f**) 650 °C.

Figure 7a–c shows SEM images of the microstructure near the fracture surface of the HIPed TiC/TC4 composite after tensile testing at 600 °C. Unlike the room-temperature case, during high-temperature tensile deformation, microcracks initiate not only in the vicinity of TiC particles but are also observed within the dual-phase matrix, albeit in smaller numbers. This indicates that the origins of fracture at high temperature include both the regions around TiC particles and the Ti matrix. Additionally, the precipitation of a finer, newly formed precipitation phase can be observed within the β-Ti matrix, resembling the secondary α phase formed during aging treatment [31]. Figure 7d–f shows SEM images of the microstructure near the fracture surface after tensile testing at 650 °C. Compared to the microstructure after testing at 600 °C, the precipitation of secondary α phase was also observed within the β phase. The number and length of microcracks within the composite matrix after testing at 650 °C significantly increased, indicating a substantial decline in the intrinsic strength of the matrix, which consequently becomes the primary origin of fracture. However, the crack tips exhibited greater blunting (higher crack tip curvature radius) compared to those after room-temperature and 600 °C testing, which aligns with the mechanical property measurements.

**Figure 8:**
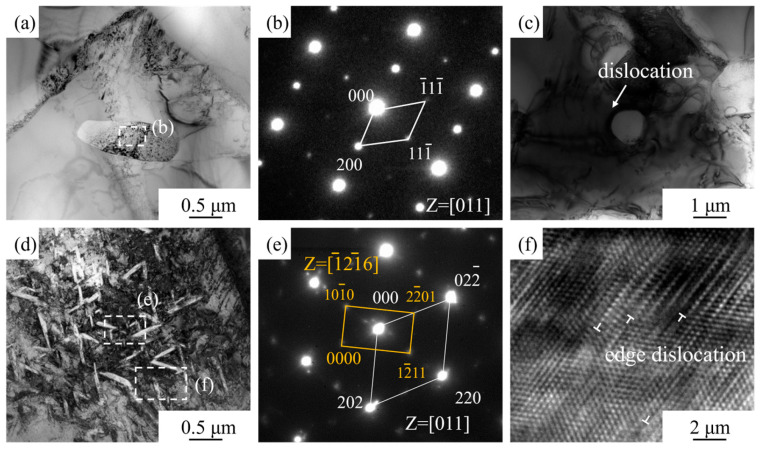
TEM images of the TiC/TC4 composite specimen after high-temperature tensile testing at 600 °C: (**a**–**c**) TiC phase; (**d**–**f**) Matrix phase.

Figure 8a,b presents a TEM bright-field image and the corresponding SAED pattern (from the dashed area) of the microstructure after high-temperature tensile fracture at 600 °C. The elliptical particle phase in Figure 8a was identified as TiC. Unlike the BF images of the room-temperature microstructure, dislocations were observed within the TiC particles after high-temperature deformation, indicating that TiC particles soften and possess a certain deformation capability at elevated temperatures. This reduces the strengthening effect of the TiC particles, leading to decreased tensile strength but improved total elongation in the composite at high temperatures. Figure 8c is a BF image of a TiC particle in another micro-region, showing a high density of dislocations surrounding it. This confirms that some TiC particles can still hinder dislocation motion at this temperature, achieving a synergistic effect of particle strengthening and load-transfer strengthening, thereby maintaining a certain strength level in the material at high temperature. Figure 8d is a BF image of the region containing the newly formed phase observed in Figure 7. This newly formed phase exhibits a fine, acicular morphology and is randomly distributed within the matrix. The SAED pattern from the dashed area (e) is shown in Figure 8e. The bright diffraction spots, indexed in white, correspond to the β-Ti phase matrix. The dark diffraction spots in the background, indexed in yellow, correspond to the α-Ti phase. This confirms that the new precipitated phase within the β-Ti matrix is the secondary nano-acicular α phase (αs). The precipitated HCP-structured αs phase can effectively pin dislocations during tensile deformation, thereby enhancing the high-temperature strength of the TiC/TC4 composite. Furthermore, a high-resolution TEM (HRTEM) image (Figure 8f) was acquired from the dashed area (f) in Figure 8d. Some edge dislocations can be observed within the β phase matrix, which is related to the easier dislocation glide in the BCC structure of the β phase, contributing to the high total elongation of the composite.

## 4. Conclusions

In this study, TiC/TC4 composites with a homogeneous microstructure were successfully fabricated via hot isostatic pressing (HIP). The evolution of mechanical properties at different temperatures and the corresponding microstructural evolution after deformation were investigated, leading to the following conclusions:

(1) The HIPed TiC/TC4 composite exhibited a typical equiaxed α+β dual-phase matrix microstructure. The TiC reinforcement particles were primarily distributed at grain boundaries, with fine TiC particles also uniformly precipitated within the grains. The tensile strength of the composite was comparable to previously reported titanium matrix composites, but its total elongation was significantly increased to 17.0 ± 0.5%. The fine matrix grains and uniformly distributed TiC particles were identified as the primary reasons for this high ductility.
(2) During room-temperature tensile deformation, the high-strength but non-plastic TiC particles readily caused dislocation pile-ups, leading to interfacial stress concentration and the initiation of microcracks, which ultimately resulted in material fracture. The predominant strengthening mechanism provided by the TiC particles at room temperature was load-transfer strengthening.
(3) At elevated temperatures, the strength of the matrix decreased, leading to the extensive initiation of microcracks within the matrix, which became the dominant fracture mechanism. At 600 °C, the composite exhibited predominantly ductile fracture, while a mixed ductile-brittle failure mode emerged at 650 °C. The TiC particles exhibited deformation compatibility at high temperatures, resulting in a diminished particle strengthening effect. Concurrently, nano-acicular αs phases precipitated from the β phase under high-temperature deformation, providing a precipitation strengthening effect.

## Data Availability

The original contributions presented in this study are included in the article. Further inquiries can be directed to the corresponding author.
